# Solid-phase synthesis and properties of stereocontrolled boranophosphate/phosphate and phosphorothioate/phosphate chimeric oligouridylates

**DOI:** 10.1098/rsos.230095

**Published:** 2023-04-12

**Authors:** Kazuki Sato, Yohei Nukaga, Takeshi Wada

**Affiliations:** Department of Medicinal and Life Sciences, Tokyo University of Science, Noda, Chiba 278-8510, Japan

**Keywords:** stereoselective synthesis, oxazaphosholidine, chimeric RNAs

## Abstract

This study describes the stereoselective synthesis of boranophosphate/phosphate (PB/PO) and phosphorothioate/phosphate (PS/PO) chimeric oligouridylates using the solid-phase method. Oxazaphospholidine monomer was used to construct the stereodefined PB and PS linkages. The study introduces modifications to oligouridylate derivatives in the intended positions with the intended stereochemistry of phosphorous atoms. Additionally, biophysical and biochemical properties of the synthesized oligomers were evaluated. Notably, it was found that a (*S*p)-PB/PO chimeric oligouridylate had higher hybridization ability than the unmodified counterpart to an unmodified oligoadenylate. This is the first report that elucidates the effect of both stereochemistry and type of *P*-modification (PB and PS) on properties of oligoribonucleotides.

## 1. Introduction

Oligonucleotide therapeutics have attracted much attention as next-generation drugs. Since discovering the RNA interference (RNAi) mechanism [1], much effort has been devoted to developing oligonucleotide therapeutics based on the RNAi mechanism [2,3]. The most commonly used modality is the small-interfering RNA (siRNA). SiRNA's gene silencing mechanism is as follows: Argonaute proteins recognize and load siRNA. Then, a strand, called the passenger strand, is cleaved and another strand, the guide strand, forms an RNA-induced silence complex (RISC) with proteins, including Argonaute proteins [4]. The RISC catalytically cleaves mRNA with the complementary sequence of the guide strand, suppressing target gene expression [5].

Five siRNA drugs, Patisiran [6], Givosiran [7], Lumasiran [8] Inclisiran [9] and Vutrisiran [10], have been approved so far and these contain partial or complete ribose modifications of siRNA structure. Additionally, the latter four have some phosphorothioate linkages at the 5′-ends of both strands and 3′-end of one strand. These chemical modifications contribute to the improvement of resistance to nuclease digestion. Therefore, the precise introduction of chemical modifications at appropriate positions is required to develop potent siRNA drugs [3].

One of the most useful tools for improving biological stability is the introduction of *P*-modified phosphate linkages into siRNAs. A phosphorothioate modification is the most prevalent strategy due to its easy access and reliable nuclease resistance [11,12]. However, the major drawback of using phosphorothioate oligonucleotides is cytotoxicity from interaction of phosphorothioate linkage with proteins [11,13]. To overcome this problem, a boranophosphate oligonucleotide, in which one of the non-bridging oxygen atoms of internucleotidic linkages is replaced with a borano group, is a promising alternative for phosphorothioate counterparts [14].

Oligoribonucleotides with boranophosphate linkages are highly nuclease-resistant and some of them show even higher silencing activity of the target gene than their unmodified counterpart [15]. Notably, boranophosphate oligonucleotides have been reported not to exhibit cytotoxicity [15,16]. These properties render boranophosphate modification an attractive option for the siRNA drug development. However, an excessive chemical modification of internucleotidic linkage results in unfavourable properties such as reduced duplex-forming ability [17] and low RNAi activity [11,15,18]. This issue is avoidable by introducing partial *P*-modifications on oligoribonucleotides, specifically, phosphate/*P*-modified phosphate chimeric ones. Additionally, *P*-modified oligonucleotides generally have diastereomers derived from phosphorous chirality. Since these diastereomers show different physico-chemical and biological behaviours [19], a stereocontrolled synthesis of *P*-modified oligonucleotides is required to obtain the desired properties.

Therefore, the stereocontrolled introduction of *P*-modifications on appropriate positions with appropriate stereochemistry leads to developing a prominent siRNA drug. We have reported the stereocontrolled synthesis of PS/PO chimeric oligodeoxyribonucleotides using an oxazaphospholidine derivative [20]. Oxazaphospholidine is a phosphoramidite derivative having a chiral auxiliary [19,21]. It offers a stereoselective condensation reaction with a hydroxy group in the presence of a non-nucleophilic acid activator to give a stereodefined phosphite linkage [22,23]. The chemical modifications on the phosphorous atom afford a *P*-modified phosphate linkage. For example, sulfurization of the phosphite intermediate gives a phosphorothioate linkage. Synthesizing PS/PO chimeric oligodeoxyribonucleotides in a stereoselective manner uses standard phosphoramidite and oxazaphospholidine monomers to construct phosphate and phosphorothioate linkages, respectively [20]. One can expect that substituting a boronation reaction for sulfurization would simply provide a boranophosphate. However, this is not the case. The boronation reaction reportedly causes serious side reactions with generally used acyl type amino protecting groups on nucleobases (adenine, cytosine and guanine) [24]. This study investigated the synthesis of PB/PO and PS/PO chimeric oligouridylates using the oxazaphospholidine approach. Since oligomers can be synthesized without protection on an uracil base, side reactions caused by boronation steps are avoidable. Although this strategy would work only for the synthesis of homouridylate derivatives, it could provide insights into the fundamental properties of chimeric oligomers.

**Scheme 1.**
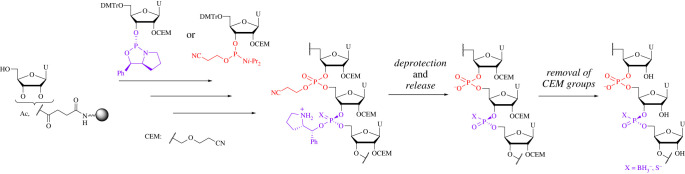
Strategy in this study.

Meanwhile, the 2′-hydroxy protecting group is necessary for the synthesis of oligoribonucleotides. The 2-Cyanoethoxymethyl (CEM) group was chosen as the 2′-hydroxy protecting group because a CEM group reportedly offers high coupling yields owing to its low steric hinderance (scheme 1) [25,26].

## 2. Results and discussion

### 2.1. Solid-phase, stereocontrolled synthesis of PB/PO and PS/PO chimeric oligomers by an automated synthesizer

**Scheme 2.**
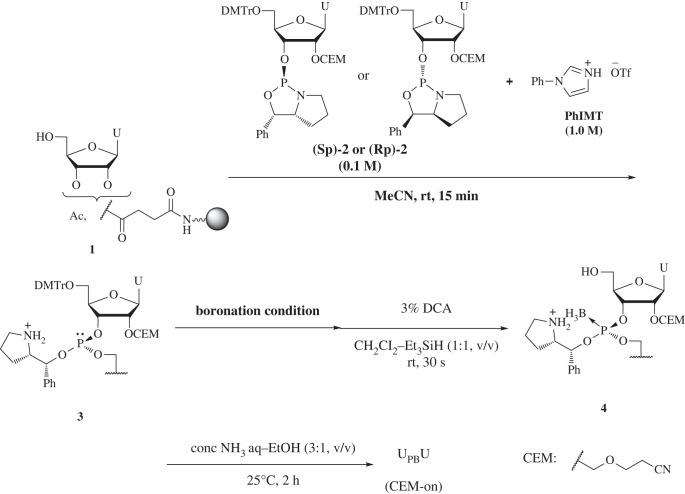
Automated synthesis of U_PB_U.

First, we attempted the synthesis of PB/PO chimeric ORNs. The boronation reaction conditions were studied to convert a phosphite intermediate to a boranophosphate. Thus, solid-phase synthesis of stereocontrolled diuridine boraphosphates was investigated using an automated synthesizer, as shown in scheme 2. A uridine bearing 5′-hydroxy group on a highly cross-linked polystyrene (HCP) [27] via a succinyl linker (**1**) was condensed with an oxazaphospholidine monomer **2** [28], whose 2′-OH was protected with a CEM group, in the presence of 1.0 M of 1-phenyl imidazolium triflate (PhIMT) to form a phosphite intermediate **3**. Then, the resultant phosphite **3** was boronated. The 5′-*O*-DMTr group was removed under acidic conditions to give an intermediate **4** in the presence of Et_3_SiH as a cation scavenger to prevent the side reaction on borano groups with DMTr cation [24,29]. Subsequent removal of the auxiliary group and cleavage of the linker by treatment with a concentrated aqueous NH_3_–EtOH (3:1, v/v) solution afforded a diuridine boranophosphate (CEM-on), and the mixture was analysed using reverse-phase high-performance liquid chromatography (RP-HPLC). The results are shown in table 1. In entry 1, (*S*p)−**2** was used as a monomer and the boronation reaction was conducted using the BH_3_·SMe_2_ complex in DMAc solvent. However, there was a large peak just after the peak derived from the product in the RP-HPLC profile (electronic supplementary material, figure S1), indicating that the BH_3_·SMe_2_ complex caused a side reaction. Using 0.05 M BH_3_·THF complex in THF instead of BH_3_·SMe_2_ complex gave the U_PB_U (CEM-on) in a high yield with good diastereoselectivity (entry 2). The (*R*p)-**2** monomer also afforded (*R*p)-U_PB_U (CEM-on) with a good HPLC yield and stereoselectivity (entry 3).

**Table 1.** Solid-phase synthesis of U_PB_U.

entry	dimer^a^	boronation conditions	HPLC yield^b^
1	(*S*p)-U_PB_U	BH_3_·SMe_2_–DMAc (6:1, v/v), 10 min	—
2	(*S*p)-U_PB_U	0.05 M BH_3_·THF/THF, 2 min	98
3	(*R*p)-U_PB_U	0.05 M BH_3_THF/THF, 2 min	95

^a^Subscript ‘PB’ = boranophosphate linkage.

^b^Determined by area ratios of RP-HPLC: U_PB_U/(U_PB_U + U).

**Scheme 3.**
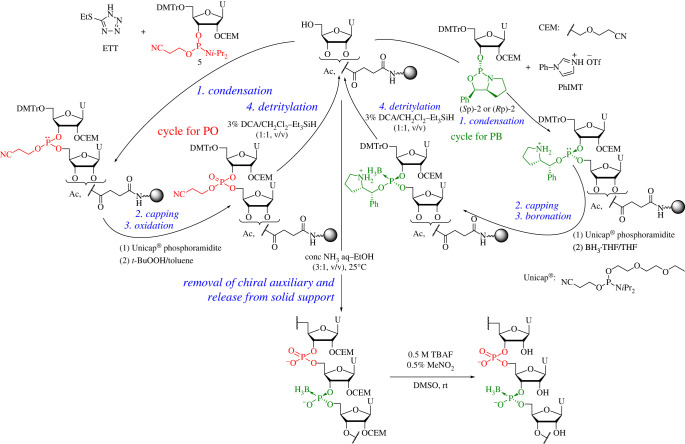
Automated solid-phase synthesis of PB/PO oligoribonucleotides.

Next, we examined the synthesis of PB/PO chimeric octamer having a stereocontrolled boranophosphate linkage. We chose *t-*butylhydroperoxide (TBHP) as an oxidizing reagent, which is necessary to create phosphate linkages because boranophosphotriester was almost inert towards TBHP in liquid-phase synthesis, as we previously reported [30]. For capping steps to block the unreacted 5′-hydroxy group, we used Unicap® phosphoramidite as a capping reagent. We have reported that CF_3_COIm is a favourable capping reagent for the stereocontrolled synthesis of PS-DNAs and RNAs using the oxazaphospholidine method [23,28]. The pyrrolidine ring of the phosphite intermediate was converted to a trifluoroacetamide moiety using CF_3_COIm. However, the reduction of trifluoroacetamide moiety upon treatment with a boronation reagent would affect the synthesis of an oligomer with boranophosphate linkages. Thus, Unicap® phosphoramidite was chosen as a capping reagent for the synthesis. CEM-on octamers, (*S*p)- and (*R*p)-U_PO_U_PO_U_PO_U_PB_U_PO_U_PO_U_PO_U were synthesized by repeated cycles consisting of the condensation, capping, boronation or oxidation and detritylation, then the removal of auxiliary groups and release from the solid support was conducted by treatment with a concentrated NH_3_ aq–EtOH (3:1, v/v) solution. To construct the phosphate linkage, we used the phosphoramidite **5** and 5-(ethylthio)-tetrazole as a monomer and activator, respectively. For the stereodefined boranophosphate linkage formation, we used the oxazaphospholidine **2** and PhIMT (scheme 3). The RP-HPLC profiles of the crude mixtures of the octamers indicated that the products had an excellent coupling yield. After the main products (CEM-on octamers) were purified using RP-HPLC, we deprotected the 2′-*O*-CEM group by treatment with tetra-n-butylammonium fluoride (TBAF) in dimethyl sulfoxide (DMSO) in the presence of 0.5% MeNO_2_ as a scavenger of acrylonitrile [31]. We successfully isolated the (*S*p)- and (*R*p)-octamers in 19% and 32% yields, respectively, based on UV absorbance value at 260 nm (table 2, entries 1 and 2). These results showed that the boranophosphotriester formed on the solid support was intact to TBHP and PB/PO chimeric oligomers were successfully synthesized using this approach. We also synthesized two types of PB/PO chimeric dodecamers having stereocontrolled boranophosphate linkages at the central and 3′-end regions, respectively, in acceptable yields (table 2, entries 3–6, 8%–11%).

**Table 2.** Synthesis of PB/PO oligoribonucleotides.

entry	oligoribonucleotide^a^	monomer	reaction time [h]		isolated yield [%]^b^	*m/z*	
			conc NH_3_aq –EtOH	TBAF		calcd	found
1	(*S*p)-U_PO_U_PO_U_PO_U_PB_U_PO_U_PO_U_PO_U	(*S*p)-**2**	3	5	19	2383.28 ([M−H]^−^)	2383.89^c^
2	(*R*p)-U_PO_U_PO_U_PO_U_PB_U_PO_U_PO_U_PO_U	(*R*p)-**2**	3	5	32	2383.28 ([M–H]^−^)	2384.26^c^
3	(*S*p)- U_PO_U_PO_U_PO_U_PO_U_PB_U_PB_U_PB_U_PO_U_PO_U_PO_U_PO_U	(*S*p)-**2**	4.5	5	11	3603.45 ([M–H]^−^)	3603.58^c^
4	(*R*p)- U_PO_U_PO_U_PO_U_PO_U_PB_U_PB_U_PB_U_PO_U_PO_U_PO_U_PO_U	(*R*p)-**2**	4	5	11	3603.45 ([M–H]^−^)	3604.42^c^
5	(*S*p)- U_PO_U_PO_U_PO_U_PO_U_PO_U_PO_U_PO_U_PO_U_PB_U_PB_U_PB_U	(*S*p)-**2**	4	9	8	800.98 ([M–5H +4Et_3_N]^5−^)	800.96^d^
6	(*R*p)- U_PO_U_PO_U_PO_U_PO_U_PO_U_PO_U_PO_U_PO_U_PB_U_PB_U_PB_U	(*R*p)-**2**	4	10	11	800.98 ([M–5H +4Et_3_N]^5−^)	800.97^d^

^a^Subscript ‘PO’ = phosphate linkage, ‘PB’ = boranophosphate linkage.

^b^Determined by UV absorbance at 260 nm of isolated oligomers.

^c^Detected by using MALDI/TOF.

^d^Detected by using ESI/Q-TOF.

**Scheme 4.**
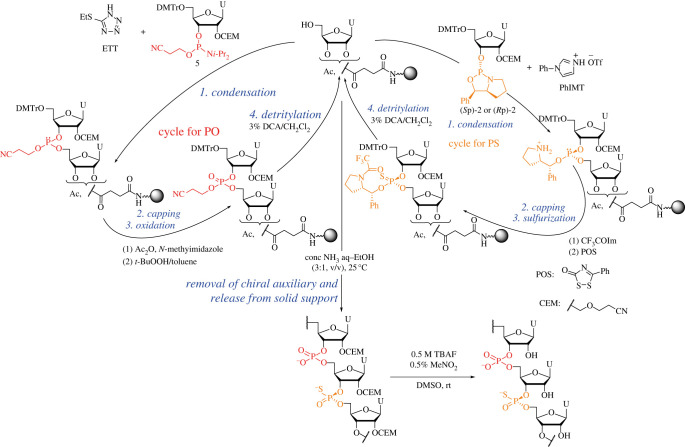
Automated solid-phase synthesis of PS/PO oligoribonucleotides.

Subsequently, the synthesis of stereocontrolled PS/PO chimeric U_12_ was attempted. The procedure that followed was that of the synthesis of PS/PO chimeric oligodeoxyribonucleotides using the oxazaphospholidine method we have previously reported [20]. To construct stereodefined phosphorothioate linkages, we condensed the oxazaphospholidine monomer (*R*p)-**2** or (*S*p)-**2** with a hydroxy group. After we conducted a capping step with CF_3_COIm and *N*-methylimidazole (NMI), we sulfurized the phosphite intermediate using 3-phenyl 1,2,4-dithiazoline-5-one (POS) [32]. To form phosphate linkages, we used the phosphoramidite **5** as a monomer, acetic anhydride (Ac_2_O) as a capping reagent and TBHP for the oxidation step. We followed the procedure for the oligomer release from a solid support and removal of the 2′-*O*-CEM group to synthesize PB/PO chimeric U_12_ (scheme 4). The PS/PO oligomers were successfully obtained in 13%–45% yields (table 3). Isolated yields of PB/PO chimeric oligomers tended to be lower than those of PS/PO counterparts. We have reported that the boronation step caused reduction of the next condensation reaction efficiency, probably due to the fact that the boronation reagent and/or the residue(s) interrupted the condensation reaction. It was found that washing a solid support with EtOH was effective to prevent retardation of the reaction [33]. Thus, isolated yields of PB/PO chimeric oligomers could be improved by the adoption of the washing step.

**Table 3.** Synthesis of PS/PO oligoribonucleotides.

entry	oligoribonucleotide^a^	monomer	reaction time [h]		isolated yield [%]^b^	*m/z*	
			conc. NH_3_aq –EtOH	TBAF		calcd	found^c^
1	(*R*p)-U_PO_U_PO_U_PO_U_PO_U_PS_U_PS_U_PS_U_PO_U_PO_U_PO_U_PO_U	(*S*p)-**2**	8	7	13	3658.28 ([M−H]^−^)	3658.55^c^
2	(*S*p)- U_PO_U_PO_U_PO_U_PO_U_PS_U_PS_U_PS_U_PO_U_PO_U_PO_U_PO_U	(*R*p)-**2**	6	7	29	810.10 ([M–11H+5Bu_4_N^+^]^6−^)	810.10^d^
3	(*R*p)-U_PO_U_PO_U_PO_U_PO_U_PO_U_PO_U_PO_U_PO_U_PS_U_PS_U_PS_U	(*S*p)-**2**	10	7	17	810.10 ([M–11H+5Bu_4_N^+^]^6−^)	810.10^d^
4	(*S*p)-U_PO_U_PO_U_PO_U_PO_U_PO_U_PO_U_PO_U_PO_U_PS_U_PS_U_PS_U	(*R*p)-**2**	4	8	45	3658.28 ([M–H]^−^)	3658.62^c^

^a^Subscript PO = phosphate linkage, ‘PS’ = phosphorothioate linkage.

^b^Determined by UV absorbance at 260 nm of isolated oligomers.

^c^Detected by using MALDI/TOF.

^d^Detected by using ESI/Q-TOF.

### 2.2. Hybridization ability

**Figure 1.**
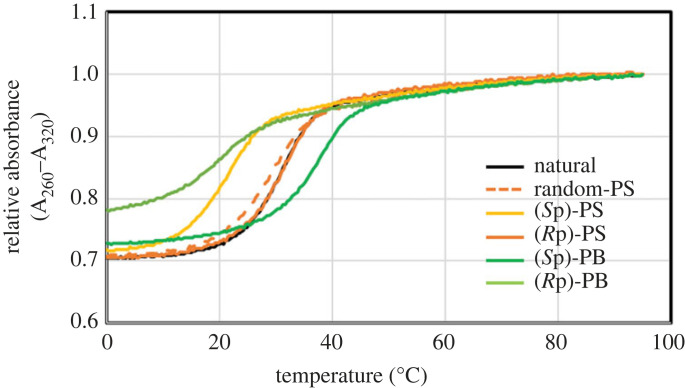
UV melting curves of duplexes of synthesized oligomers with r(A_PO_)_11_A in the presence of 100 mM Na_2_HPO_4_–NaH_2_PO_4_ and 1 M NaCl (pH 7.0).

Next, we moved to the evaluation of the obtained oligomers. We annealed each stereocontrolled PB/PO and PS/PO chimeric dodecauridylate having *P*-modified linkages in their central region with an equal amount of dodecaadenylate in a buffer solution, evaluating the *T*_m_ value with a thermal denaturation test. Table 4 shows the result. Because some of the melting curves were not sigmoidal in the presence of 0.1 M NaCl and 10 mM Na_2_HPO_4_–NaH_2_PO_4_, we could not determine the *T*_m_ values of all duplexes (electronic supplementary material, figure S8). Thus, *T*_m_ values were calculated from the results obtained from the experiments with a higher salt concentration (1 M NaCl and 0.1 M Na_2_HPO_4_–NaH_2_PO_4_, figure 1). Compared to the unmodified U_12_, stereorandom PS/PO chimeric U_12_ showed lower hybridization ability. As for stereocontrolled counterparts, (*S*p)-PS/PO and (*R*p)-PB/PO-ORNs had reduced duplex-forming ability, whereas (*R*p)-PS/PO-ORN showed comparable ability. Notably, (*S*p)-PB/PO chimeric U_12_ formed a marked stable duplex. This result agrees well with our previous report that an all-(*S*p)-PB 2′-*O*-Me decauridylate formed a more stable duplex with a complementary ORN than an unmodified counterpart, whereas the *T*_m_ value of an all-(*R*p)-PB 2′-*O*-Me decauridylate and complementary ORN could not be determined owing to the instability of duplex [34]. From the observation of melting curves, it was suggested that the duplex of (*R*p)-PB/PO chimeric U_12_ and A_12_ had a small hyperchromicity compared to the other duplexes. We attributed the phenomenon to the low thermal stability of the duplex. A part of the duplex might be dissociated even at 0°C. From these results, the order of hybridization ability of dodecamers was found to be as follows: (*S*p)-PB/PO > (*R*p)-PS/PO ≈ unmodified > (*S*p)-PS/PO > (*R*p)-PB/PO.

**Table 4.** *T*_m_ values of 2 µM duplexes of dodecauridine derivatives with rA_12_.

entry	oligoribonucleotide	*T*_m_ [°C]*^a^*	Δ*T*_m_ [°C]*^b^*
1	U_PO_U_PO_U_PO_U_PO_U_PO_U_PO_U_PO_U_PO_U_PO_U_PO_U_PO_U	30.0 ± 0.1	—
2	stereorandom- U_PO_U_PO_U_PO_U_PO_U_PS_U_PS_U_PS_U_PO_U_PO_U_PO_U_PO_U	28.4 ± 0.4	−1.6 ± 0.3
3	(*S*p)-U_PO_U_PO_U_PO_U_PO_U_PS_U_PS_U_PS_U_PO_U_PO_U_PO_U_PO_U	20.8 ± 0.7	−9.2 ± 0.6
4	(*R*p)-U_PO_U_PO_U_PO_U_PO_U_PS_U_PS_U_PS_U_PO_U_PO_U_PO_U_PO_U	30.9 ± 0.3	+0.8 ± 0.3
5	(*S*p)-U_PO_U_PO_U_PO_U_PO_U_PB_U_PB_U_PB_U_PO_U_PO_U_PO_U_PO_U	36.5 ± 0.7	+6.5 ± 0.7
6	(*R*p)-U_PO_U_PO_U_PO_U_PO_U_PB_U_PB_U_PB_U_PO_U_PO_U_PO_U_PO_U	20.6 ± 1.8	−9.4 ± 1.9

*^a^*Avarage values of the experiments in triplicate. Error bars denote standard deviation. buffer conditions: 1 M NaCl and 0.1 M Na_2_HPO_4_–NaH_2_PO_4_. (pH 7.0).

*^b^*Difference in *T*_m_ value compared to unmodified counterpart.

To gain further insights into the effect of chemical modifications on duplex formation, the thermodynamic parameters of duplex formation were calculated by denaturation tests at varied duplex concentrations. Unmodified, (*R*p)-PS/PO and (*S*p)-PB/PO chimeric dodecauridylates, which formed relatively stable duplexes, were used for the experiments and the results are shown in table 5 and electronic supplementary material, figure S9. Compared to the duplex of unmodified dodecauridylate and dodecaadenylaye, the Δ*H* and Δ*S* values of duplex formation of (*R*p)-PS/PO chimeric dodecauridylate and the complementary strand was clearly lower. On the other hand, Δ*H* and Δ*S* values of (*S*p)-PB/PO chimeric dodecauridylate and dodecaadenylaye were comparable and slightly higher than those of the unmodified duplex, respectively. These results suggested that introduction of (*R*p)-PS modification led to an enthalpy gain upon duplex formation whereas (*S*p)-PB modification resulted in a reduced entropy loss. The detailed study must be conducted with oligomers bearing four nucleobases.

**Table 5.** *T*_m_ values of duplexes of dodecauridine derivatives with rA_12_ at the concentration of 1–16 µM and thermodynamic parameters of duplex formation.

entry	oligoribonucleotide	*T*_m_ [°C] value at concentration					Δ*H*° (kcal mol^−1^)	Δ*S*° (cal mol^−1^ K^−1^)	Δ*G*° at 37°C (kcal mol^−1^)
		1 µM	2 µM	4 µM	8 µM	16 µM			
1	U_PO_U_PO_U_PO_U_PO_U_PO_U_PO_ U_PO_U_PO_U_PO_U_PO_U_PO_U	27.6	30.1	33.2	35.4	37.6	−50.8	−139.3	−7.8
2	(*R*p)-U_PO_U_PO_U_PO_U_PO_U_PS_ U_PS_U_PS_U_PO_U_PO_U_PO_U_PO_U	28.8	31.6	33.9	36.0	38.5	−54.5	−150.2	−7.9
3	(*S*p)-U_PO_U_PO_U_PO_U_PO_U_PB_ U_PB_U_PB_U_PO_U_PO_U_PO_U_PO_U	33.1	35.8	38.7	40.7	43.8	−50.9	−135.9	−8.7

### 2.3. Nuclease resistance

**Figure 2.**
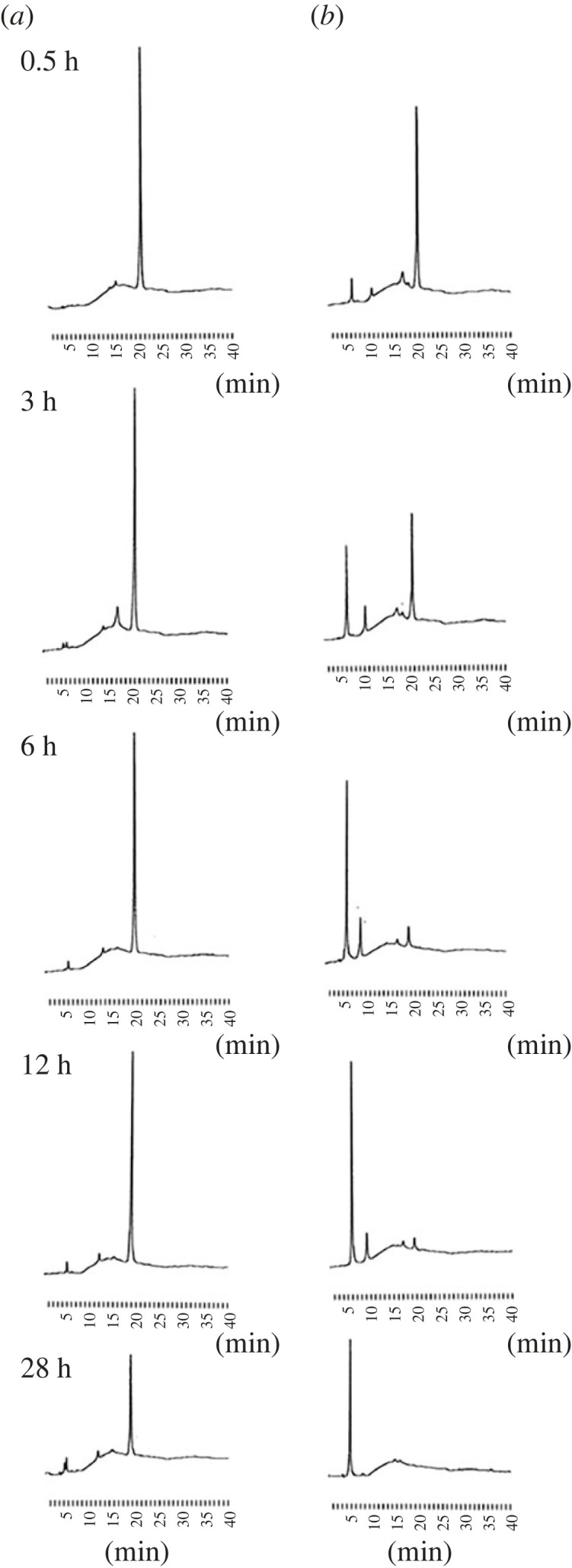
RP-HPLC profiles of (*a*) (*R*p)-U_PB_U (*b*) (*S*p)-U_PB_U after the treatment of SVPDE from *Crotalus adamanteus*. RP-HPLC was performed with a linear gradient of 0%–20% MeCN in 0.1 M TEAA buffer for 40 min at 55°C, detection at 260 nm, a flow rate of 0.5 ml min^−1^ and a C18 column.

Subsequently, we investigated the nuclease resistance of synthesized oligomers using snake venom phosphodiesterase (SVPDE) from *Crotalus adamanteus*, a representative 3′-exonuclease [35]. First, we treated diastereomers (*S*p), (*R*p)-U_PB_U with SVPDE for designated times and then divided and analysed a portion of the solution using RP-HPLC. The RP-HPLC profiles clearly showed that (*R*p)-U_PB_U was stable under the reaction conditions, whereas (*S*p)-U_PB_U was gradually cleaved (figure 2). This result suggested that the (*R*p)-counterpart had higher resistance to SVPDE digestion and did not contradict previous reports [36], suggesting that these dimers were obtained with intended stereochemistry.

**Figure 3.**
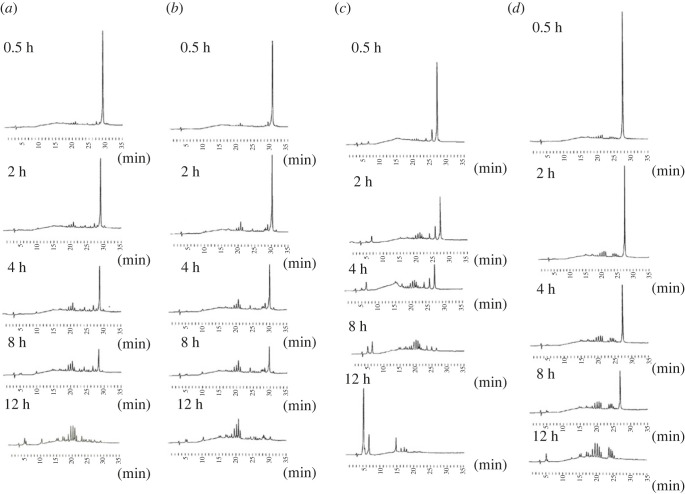
RP-HPLC profiles of (*a*) (*S*p)- U_PO_U_PO_U_PO_U_PO_U_PO_U_PO_U_PO_U_PO_U_PB_U_PB_U_PB_U, (*b*) (*R*p)- U_PO_U_PO_U_PO_U_PO_U_PO_U_PO_U_PO_U_PO_U_PB_U_PB_U_PB_U, (*c*) (*R*p)- U_PO_U_PO_U_PO_U_PO_U_PO_U_PO_U_PO_U_PO_U_PS_U_PS_U_PS_U and (*d*) (*S*p)- U_PO_U_PO_U_PO_U_PO_U_PO_U_PO_U_PO_U_PO_U_PS_U_PS_U_PS_U after the treatment of SVPDE from *Crotalus adamanteus*. RP-HPLC was performed with a linear gradient of 0%–24% MeCN in 0.1 M TEAA buffer for 45 min at 55°C, detection at 260 nm, a flow rate of 0.5 ml min^−1^ and a C18 column.

Next, we treated PB/PO and PS/PO chimeric oligomers having *P*-modified linkages at 3′-ends with SVPDE to investigate the effect of the type of modification and configuration of phosphorous atoms on nuclease resistance. Figure 3 and electronic supplementary material, figure S10 show the RP-HPLC profiles of the reaction mixtures. The unmodified dodecamer was swiftly cleaved using SVPDE (electronic supplementary material, figure S10). However, the stereocontrolled chimeric oligomers were not completely cleaved within 4 h. Among the stereocontrolled chimeric oligomers, (*R*p)-PS/PO U_12_ degraded the most rapidly, followed by its (*R*p) and (*S*p)-PB/PO counterparts. (*S*p)-PS/PO U_12_ seemed to be the most resistant to the SVPDE digestion. The effect of phosphorous configuration on nuclease resistance was apparent for PS/PO chimeric U_12_, whereas (*R*p) and (*S*p)-PB/PO oligomers showed similar behaviour.

## 3. Conclusion

We have developed an efficient method for synthesizing PB/PO and PS/PO chimeric oligouridylate derivatives in a stereoselective manner. Concurrent usage of phosphoramidite and oxazaphospholidine monomers enabled the construction of both phosphate and stereocontrolled *P*-modified linkages. We used Unicap® phosphoramidite for the capping steps in the synthesis of PB/PO-ORNs to suppress side reactions. We also evaluated the biophysical and biochemical properties of the resultant oligomers. The following order was elucidated:

hybridization ability to complementary ORN: (*S*p)-PB/PO > (*R*p)-PS/PO ≈ unmodified > (*S*p)-PS/PO > (*R*p)-PB/PO

3′-exonuclease resistance: (*S*p)-PS/PO > (*R*p)-PB/PO ≈ (*S*p)-PB/PO > (*R*p)-PS/PO > unmodified.

Although we have shown a few synthetic examples, the method enables the introduction of boranophosphate or phosphorothioate linkages at intended positions with high stereoselectivity. Furthermore, insights obtained in this study would be valuable for the rational design of chimeric oligoribonucleotides with improved properties.

## 4. Experimental section

### 4.1. General information

Synthesized dimers were analysed using RP-HPLC. Synthesized oligomers (octamers and dodecamers) were analysed and purified using reversed-phase HPLC and identified by MALDI or ESI mass spectroscopy. Isolated yields of oligomers were estimated by measuring the UV–Vis spectra using the following molar absorption constant at 260 nm (octamer: *ε* = 80 280 l mol^−1^ cm^−1^; dodecamer: *ε* = 120 320 l mol^−1^ cm^−1^).

### 4.2. A general procedure for the automated stereocontrolled synthesis of dinucleoside boranophosphates

An automated solid-phase synthesis of (*S*p) and (*R*p)-U_PB_U was conducted following the procedure shown in table 6 using 5′-*O*-DMTr-uridine loaded HCP via a succinyl linker (0.2 µmol, 26.6 µmol g^−1^). After the synthesis, we treated the HCP with concentrated aqueous NH_3_–EtOH (3:1, v/v) at 25°C for 3 h, filtered and washed with EtOH. The filtrate and washings were combined and concentrated under reduced pressure. The residue was analysed using RP-HPLC, conducted with a linear gradient of 0%–30% MeCN in a 0.1 M triethylammonium acetate (TEAA) buffer (pH 7.0) at 30°C for 60 min with a flow rate of 0.5 ml min^−1^ in a C18 column.

**Table 6.** Procedure for the synthesis of U_PB_U.

entry	operation	reagents and solvents	time
1	detritylation	3% (v/v) DCA in dry CH_2_Cl_2_–Et_3_SiH (1:1, v/v)	30 s
2	washing	dry MeCN	—
3	condensation	0.1 M (*S*p) or (*R*p)-**2**, 1 M PhIMT in dry MeCN	15 min
4	washing	dry MeCN	
5	boronation	BH_3_·SMe_2_–DMAc (1:6, v/v) , 10 min or 0.05 M BH_3_·THF in dry THF, 2 min	10 or 2 min
6	washing	dry MeCN	—
7	detritylation	3% (v/v) DCA in dry CH_2_Cl_2_–Et_3_SiH (1:1, v/v)	30 s
8	washing	dry MeCN	—

### 4.3. A general procedure for the automated stereocontrolled synthesis of octamers and dodecamers

An automated solid-phase synthesis of (*S*p) and (*R*p)-octamers and dodecamers was conducted following the procedure shown in table 7 (PB/PO-ORN) or table 8 (PS/PO-ORN) using 5′-O-DMTr-uridine loaded HCP via a succinyl linker (17.5 µmol g^−1^, 0.2 µmol). After the designed length was achieved, the HCP was treated with concentrated aqueous NH_3_–EtOH (3:1, v/v) at 25°C for 3–10 h, filtered and washed with EtOH. The filtrate and washings were combined and concentrated under reduced pressure. The residue was analysed and purified using RP-HPLC, conducted with a linear gradient of 0%–40% MeCN in a 0.1 M TEAA buffer (pH 7.0) at 55°C for 75 min with a flow rate of 0.5 ml min^−1^ in a C18 column.

**Table 7.** Procedure for the synthesis of stereocontrolled PB/PO octamers and dodecamers.

entry	operation	cycle for PO		cycle for PB	
		reagents and conditions	time	reagents and conditions	time
1	detritylation	3% (v/v) DCA in dry CH_2_Cl_2_–Et_3_SiH (1:1, v/v)	30 s	3% (v/v) DCA in dry CH_2_Cl_2_–Et_3_SiH (1:1, v/v)	30 s
2	washing	dry MeCN	—	dry MeCN	—
3	condensation	0.1 M **5**, 0.25 M ETT in dry MeCN	3 min	0.1 M (*S*p) or (*R*p)-**2**, 1 M PhIMT in dry MeCN	15 min
4	washing	dry MeCN	—	dry MeCN	—
5	capping	0.07 M Unicap®, 0.25 M ETT in dry MeCN	10 s	0.07 M Unicap®, 0.25 M ETT in dry MeCN	10 s
6	washing	dry MeCN	—	dry MeCN	—
7	oxidation/boronation	1 M TBHP in toluene	30 s	0.05 M BH_3_·THF in dry THF	2 min
8	washing	dry MeCN	—	dry MeCN	—

**Table 8.** Procedure for the synthesis of stereocontrolled PS/PO dodecamers.

entry	operation	cycle for PO		cycle for PS	
		reagents and conditions	time	reagents and conditions	time
1	detritylation	3% (v/v) DCA in dry CH_2_Cl_2_	30 s	3% (v/v) DCA in dry CH_2_Cl_2_	30 s
2	washing	dry MeCN	—	dry MeCN	—
3	condensation	0.1 M **5**, 0.25 M ETT in dry MeCN	3 min	0.1 M (*S*p) or (*R*p)-**2**, 1 M PhIMT in dry MeCN	15 min
4	washing	dry MeCN	—	dry MeCN	—
5	capping	Ac_2_O, 16% NMI in dry THF	40 s	0.5 M CF_3_COIm, 16% NMI in dry THF	40 s
6	washing	dry MeCN	—	dry MeCN	—
7	oxidation/boronation	1 M TBHP in toluene	30 s	0.3 M POS in dry MeCN	8 min
8	washing	dry MeCN	—	dry MeCN	—

### 4.4. A general procedure for the removal of the 2′-*O*-CEM group

The purified octamer or dodecamer bearing CEM group on the 2′ positions was treated with 0.5 M TBAF solution in dry DMSO containing 0.5% MeNO_2_ (400 µl, v/v). After 5–10 h, the reaction was quenched by the addition of 40 ml 0.1 M TEAB buffer (pH 7). The mixture was desalted by C18 cartridge. The cartridge was washed with 5% MeCN and the desired oligomer was eluted by 40% MeCN. The solution was lyophilized. The residue was analysed and purified by RP-HPLC, which was performed using a linear gradient of 0%–40% MeCN for 75 min in 0.1 M TEAA buffer (pH 7.0).

### 4.5. Thermal denaturation test

A solution containing pairs of complementary strands (2.0 µM, 0.3 nmol each) and 100 mM NaCl in a 10 mM NaH_2_PO_4_–Na_2_HPO_4_ buffer or 1 M NaCl in 100 mM NaH_2_PO_4_–Na_2_HPO_4_ buffer (pH 7.0) was heated for 10 min at 90°C and cooled to 0°C at a rate of 1.0°C/min and then left at 0°C for 30 min. Denaturation tests were conducted in a 1 cm path length quartz cell. The temperatures at the crossing points of the melting curves and the median lines of the low- and high-temperature baselines were calculated as *T*_m_ values. The three independent tests were conducted and the average values along with standard deviations were calculated.

### 4.6. Evaluation of thermodynamic parameters for the duplex formation

A solution containing pairs of complementary strands (16 µM and 2.4 nmol each) and 1 M NaCl in a 100 mM NaH_2_PO_4_–Na_2_HPO_4_ buffer (pH 7.0) was heated for 10 min at 90°C and cooled to 0°C at a rate of 1.0°C/min and then left at 0°C for 30 min. The diluted duplex solutions (8, 4, 2, 1 µM duplex) were also prepared by diluting the solution by a 1 M NaCl aqueous solution in a 100 mM NaH_2_PO_4_–Na_2_HPO_4_ buffer (pH 7.0). Denaturation tests were conducted in a 1 cm path length quartz cell. The temperatures at the crossing points of the melting curves and the median lines of the low- and high-temperature baselines were calculated as *T*_m_ values. The Δ*H*° and Δ*S*° values were calculated from the following equation [37].

$$\frac{1}{T_{m}}=\frac{R}{\Delta H}ln\left(\frac{C}{4}\right)+\frac{\Delta S}{\Delta H}$$

where R: gas constant, C: concentration of duplex

### 4.7. Enzymatic digestion of *P*-stereodefined PB-diuridylate with SVPDE

In the enzymatic digestion experiments, 5.0 nmol (50 µM) of each dimer was treated with SVPDE from Crotalus adamanteus (0.3 U) in a 5 mM Tris-HCl buffer (100 µl, pH 9.3) containing 0.1 mM MgCl_2_, 0.01 mM ZnCl_2_ and 0.1 mM spermidine at 37°C. The reactions were conducted for 0.5, 3, 6, 12 and 28 h. After the designated time, a part of the reation mixture was divided and a 0.1 M TEAA buffer (100 µl) was added to the mixture, and the mixture was heated to 100°C for 1 min to denature the enzyme. The mixture was analysed using RP-HPLC.

### 4.8. Enzymatic digestion of *P*-stereodefined PB/PO, PS/PO and unmodified dodecauridylate with snake venom phosphodiesterase

In the enzymatic digestion experiments, 1.0 nmol (10 µM) of each oligomer was treated with SVPDE from Crotalus adamanteus (2 × 10^–2^ U) in a 5 mM Tris-HCl buffer (100 µl, pH 9.3) containing 0.1 mM MgCl_2_, 0.01 mM ZnCl_2_ and 0.1 mM spermidine at 37°C. The reactions were conducted for 0.5, 2, 4, 8 and 12 h. After the designated time, a part of the reation mixture was divided, a 0.1 M TEAA buffer (100 µl) was added to the mixture, and the mixture was heated to 100°C for 1 min to denature the enzyme. The mixture was analysed using RP-HPLC.

## Data Availability

The experimental procedures and datasets supporting this article have been uploaded as part of the electronic supplementary material [38].
